# Changes in bio‐accessibility, polyphenol profile and antioxidants of quinoa and djulis sprouts during in vitro simulated gastrointestinal digestion

**DOI:** 10.1002/fsn3.1718

**Published:** 2020-06-15

**Authors:** Qinping Zhang, Bao Xing, Menghan Sun, Bangwei Zhou, Guixing Ren, Peiyou Qin

**Affiliations:** ^1^ Institute of Crop Sciences Chinese Academy of Agricultural Sciences Beijing China; ^2^ School of Pharmacy and Bioengineering Chengdu University Chengdu China; ^3^ Key Laboratory of Vegetation Ecology Ministry of Education Institute of Grassland Sciences Northeast Normal University Jilin China

**Keywords:** antioxidant activity, djulis, in vitro digestion, polyphenols, quinoa, sprouts

## Abstract

This study aimed to evaluate the bio‐accessibility of the phenolics and flavonoid, the polyphenolic profile and the antioxidant activity of sprouts obtained from four different quinoa genotypes and one djulis cultivar during in vitro gastrointestinal digestion. Compared to their content in sprouts, the bioavailable phenolics after the oral phase, the gastric phase, the intestinal phase, and in the dialyzable fraction were in the ranges of 45.7%–63.5%, 87.6%–116.7%, 89.6%–124.5%, and 7.4%–10.9%, respectively. The trend in flavonoid bio‐accessibility was similar to the polyphenols. The dialyzable flavonoid recoveries varied between 4.2% and 12.4%. Correspondingly, the free radical scavenging activity of the dialyzable phase decreased significantly from 84.7% to 96.5%. The main phenolic acids were vanillic acid, caffeic acid, and syringic acid during digestion. The results suggest that gastrointestinal digestion greatly affected the absorption of polyphenols and flavonoid of quinoa and djulis sprouts, as well as their antioxidant capacity.

## Graphical abstract

In this study, we preliminary studied the bio‐accessibility of phenolic compounds and antioxidant activities from quinoa and djulis sprouts by in vitro simulated gastrointestinal digestion. Digestion significantly decreased the absorption of polyphenols and flavonoid, as well as the antioxidant activity.

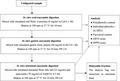

## 1 INTRODUCTION

Quinoa (*Chenopodium quinoa* Willd.), an annual dicotyledonous plant in the Amaranthaceae family, originates from the Andean region of South America. It has attracted much attention in recent times for its high nutritional value and resistance to abiotic stresses (Ramesh, Devi, Gopinath, & Praveen, 2019). As a multipurpose crop, quinoa has been cultivated as a leafy vegetable and subsidiary grain for both human consumption and animal feed in different parts of the world (Villa, Russo, Kerbab, Landi, & Rastrelli, 2014). Djulis (*Chenopodium formosanum* Koidz) is also a pseudocereal crop of the Amaranthaceae family with a close botanical similarity to quinoa and is an indigenous plant to Taiwan that has been consumed for many years. Its beneficial nutritional and health attributes have been highlighted recently (Huang, Chu, Sridhar, & Tsai, 2019; Narkprasom, Wang, Hsiao, & Tsai, 2012). The current global interest in healthier lifestyles has increased market demands for leafy vegetables that are rich in bioactive compounds. Previous studies have shown that quinoa sprouts are a good source of total phenolic compounds with high antioxidant activity that would be suitable for human consumption (Paśko et al., 2009; Paśko, Sajewicz, Gorinstein, & Zachwieja, 2008). The leaves of quinoa possess a broad spectrum of biological activities, such as antioxidant, anti‐inflammatory, and cytostatic effects (Chen et al., 2017; Gawlik‐Dziki et al., 2013). Therefore, quinoa and djulis could be cultivated as nutraceutically valuable green leafy vegetables due to the high nutraceutical potential of their leaves and sprouts.

Polyphenols are being widely studied for their potential positive effects in foods. Phenolic compounds are the major low molecular weight bioactive components usually found in plants, and have shown various positive bioactivities, such as antioxidant, anti‐inflammatory and anticarcinogenic properties (Bellion et al., 2010; Chai et al., 2020; Gawlik‐Dziki, 2012; Kim, Hwang, Kim, & Choi, 2020; Shen et al., 2019). Although the level of phenolic compounds may be obtained from composition tables, the total phenolic compounds and other natural antioxidants present in food are not necessarily direct predictors of their real effects on human health (Gunathilake, Ranaweera, & Rupasinghe, 2018; Pellegrini et al., 2017). In order to be absorbed and effective within the human body, phenolic compounds must be released from the plant tissue matrix and modified in the gastrointestinal tract and then absorbed in the gut up to a certain level (Parada & Aguilera, 2007). Bio‐accessibility is defined as the proportions of phenolic compounds that are available for absorption during gastrointestinal digestion (Palafox‐Carlos, Ayala‐Zavala, & González‐Aguilar, 2011). Bioactive compounds will exert health benefits if they remain available for absorption after all the phases of the gastrointestinal digestion process are finished. Moreover, there are a number of factors associated with the bio‐accessibility of phenolic acids, including proteins, carbohydrates, and lipids, which surround polyphenols inside the gastrointestinal tract (Gong, Chi, Zhang, Wang, & Sun, 2019; Jakobek, 2015; Zhu, 2015). Among the methods used to determine the bio‐accessibility of bioactive compounds, simulated gastrointestinal digestion is considered a valid and rapid alternative that does not possess the ethical restrictions of in vivo methods (Pellegrini et al., 2017).

However, there have been no systematic studies on the bio‐accessibility of phenolic compounds from quinoa and djulis sprouts during digestion. Understanding the factors that affect the bio‐accessibility and bioavailability of bioactive substance is important for evaluating their biological significance and efficacy as functional food ingredients. Therefore, this study aimed to investigate the effect of simulated in vitro gastrointestinal digestion on the recovery and bio‐accessibility indexes of polyphenols and flavonoids, the polyphenolic profile and the antioxidant activity of sprouts from quinoa and djulis.

## 2 MATERIALS AND METHODS

### 2.1 Chemicals and reagents

Rutin, gallic acid, α‐amylase, bile salts (a mixture of sodium cholate and sodium deoxycholate), pepsin from pig gastric mucosa, pancreatin from pig pancreas, and 1,1‐diphenyl‐2‐picrylhydrazyl (DPPH) radical were purchased from Sigma‐Aldrich. HPLC grade acetonitrile, trifluoroacetic acid (TFA, 99%), methanol, NaHCO_3,_ and other analytical reagents were purchased from Beijing Chemical Works.

### 2.2 Plant material and sample preparation

Four different quinoa genotypes and one djulis cultivar were analyzed in this study. The varieties chosen were: Red quinoa (RQ), collected from Yunnan province; White quinoa (WQ), collected Qinghai province; Mengli 1 (Gray quinoa, GQ), which is a new variety from Inner Mongolia; Altiplano from Peru, which was collected from Xinjiang province (AQ); and One djulis cultivar collected from Shandong province (DK). The experiment was conducted in a greenhouse in Qingdao (Shandong, China) in November 2018. Aerial parts of fresh samples were harvested after 30 days of growth. The sprouts were dried at 70°C, and then ground into powder before they were stored at −18°C until further use.

### 2.3 Extraction procedures

Samples (0.2 g each) were extracted with 40 ml of 80% methanol at 70°C for 2 hr in a water shaker with agitation (120 rpm) and centrifuged at 4,000 rpm for 10 min. The supernatants were subsequently filtered through 0.22 μm filter paper and stored at −18°C until further use.

### 2.4 Gastrointestinal digestion

**FIGURE 1:**
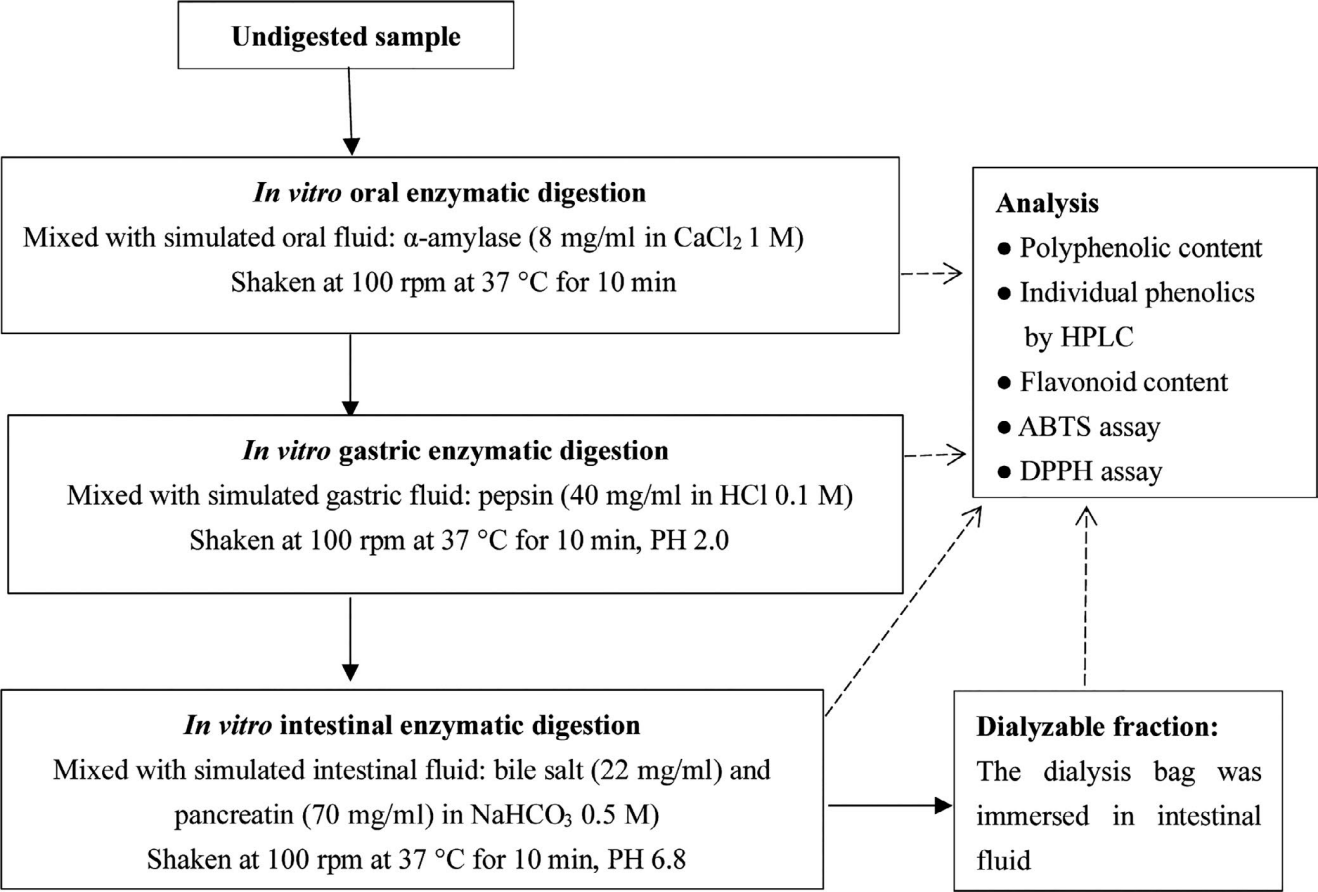
Flow diagram of the in vitro gastrointestinal digestion procedure

The in vitro digestion model was performed as previously described (Gunathilake et al., 2018; Pellegrini et al., 2017) with some modification and the steps are shown in Figure 1. Approximately, 10 g of sample was mixed with 200 ml distilled water and placed in a 500 ml beaker. The oral phase was simulated by adding 1.2 ml of 8 mg/ml salivary α‐amylase dissolved in CaCl_2_ (1 mmol/L, pH 7.0) solution. The mixture was incubated in a water bath (37°C) with agitation (100 rpm) for 10 min, protected from light. After the oral digestion, 10 ml of suspension was transferred to a test tube for analysis of bioactive compounds and antioxidant activity, and the reactions were stopped by cooling the test tubes in ice. For the gastric phase, the pH of each sample solution was adjusted to 2.0 with a 6 M HCl solution and 5 ml of pepsin solution (40 mg/ml in 0.1 M HCl) was added, followed by incubation (1 hr, 37°C, 100 rpm). After the gastric digestion, the 10 ml suspension was transferred to a test tube and immediately stopped reaction by cooling in ice. For the intestinal phase with dialysis, segments of cellulose membrane dialysis tubing (flat width: 25 mm, MWCO 14,000 Da) were cut into 15.0 cm lengths, washed with 0.9% NaCl solution, and then one end of each segment was sealed with clips. The dialysis bags are a simplified model that represents the epithelial barrier. The prepared dialysis bags were filled with 5 ml of 0.9% NaCl and 5 ml of 0.5 M NaHCO_3_ without leaving any air bubbles inside. Then the other ends of the dialysis bags were sealed with clips and immediately immersed into the appropriate gastric digest, followed by incubation (45 min, 37°C, 100 rpm). After this step, the digest transitioned from the gastric phase to the intestinal phase. The pH of the mixture was adjusted to 6.8 with 6 M NaOH, followed by the addition of 10 ml pancreatin and bile mixture (22 mg/ml pancreatin and 70 mg/ml bile extract dissolved in 0.5 M NaHCO_3_). The mixture was incubated for 2 hr in a water bath (37°C) with agitation (100 rpm). After incubation, the 10 ml suspension was transferred and stopped reaction in ice. The dialysis bags were carefully separated from the beakers and rinsed with water. The solution in each of the dialysis bags was transferred to a test tube and diluted to a final volume of 20 ml with an addition of 0.9% NaCl and stopped reaction in ice. The proteins and polysaccharides from each of the digested phases were precipitated the suspensions by adding acetone to each test tube make an 80% solution. Then, the resulting suspension was centrifuged to remove sediment and the supernate was stored at −18°C until analysis.

### 2.5 Analysis of phenolic compounds

Total polyphenol content (TPC) was determined using the Folin–Ciocalteu method described by our laboratory (Qin, Wu, Yao, & Ren, 2013) and the results expressed as milligrams of gallic acid equivalents (GAE) per gram of dry weight of the sample.

The individual phenolic acid profiles were analyzed by HPLC (Shimadzu LC‐20A series HPLC), as described previously (Zhang, Wang, Yao, Yan, & He, 2012) with a slight modification. The analytical column was a Thermo Syncronis C18 column (250 × 4.60 mm, 5 mm), and the wavelength of the UV detector was set at 260 and 280 nm. The mobile phase consisted of a mixture of 0.05% trifluoroacetic acid (solvent A) and 30% acetonitrile, 10% methanol, and 0.05% trifluoroacetic acid (solvent B). The gradient elution was as follows: 0–10 min, 20%–30% B; 10–18 min, 30%–40% B; 18–35 min, 40%–70% B; 35–40 min, 70% B; 40–45 min, 70%–72% B; 45–48 min, 72%–100% B; 48–52 min, 100% B; 52–55 min, 100%–20% B; and 55–60 min, 20% B. The flow rate was 1.0 ml/min, and the injection volume was 10 μL.

### 2.6 Analysis of flavonoid compounds

Total flavonoid content (TFC) was determined using the aluminum chloride colorimetric method as described by Sarker and Oba (2018) and the results were expressed as milligrams of rutin equivalents (RE) per gram of dry weight of the sample.

### 2.7 Antioxidant activities

The DPPH radical scavenging assay and the ABTS radical scavenging assay were used for the evaluation of antioxidant activities according our previous study (Yao et al., 2013).

### 2.8 Statistical analysis

Results are expressed as means ± *SD* of triplicate determinations. All calculations were performed using SPSS (Statistics for Social Science) version 17.0. Statistical significance was established at probability values of <0.05.

## 3 RESULTS AND DISCUSSION

### 3.1 Effect of in vitro gastrointestinal digestion on the TPC

**FIGURE 2:**
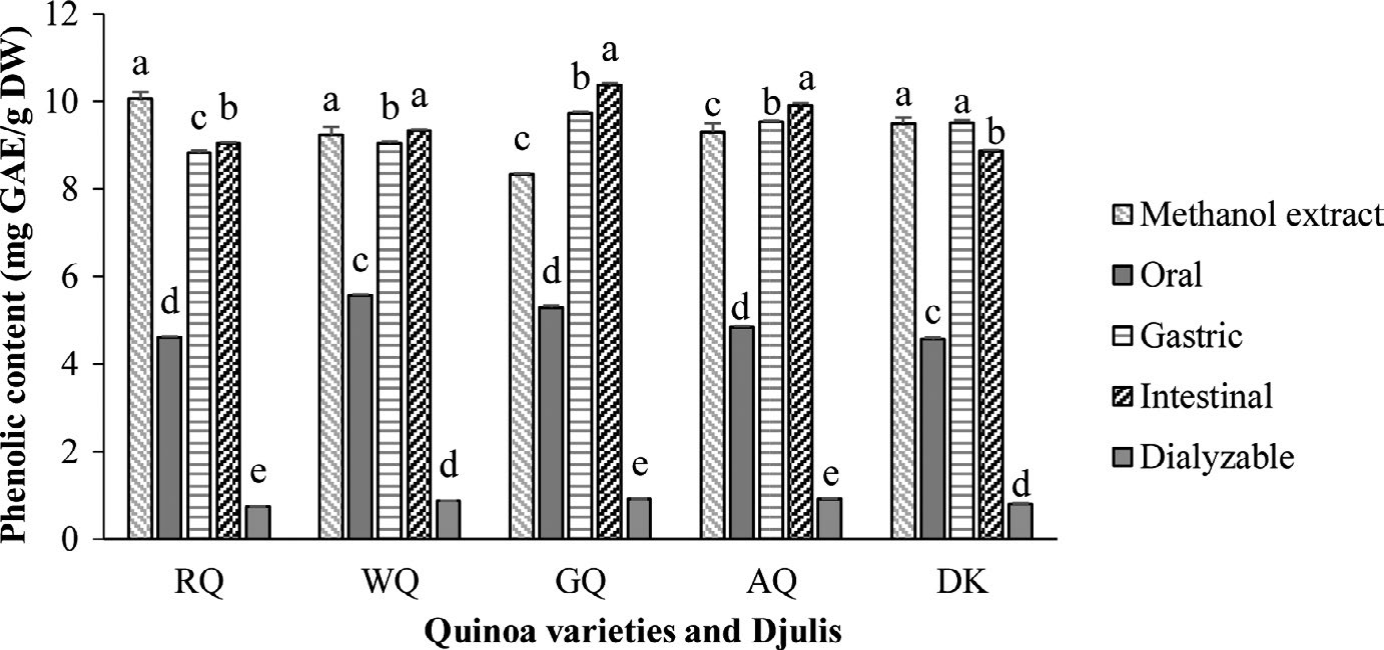
Total phenolic content of sprouts subjected to simulated in vitro oral, gastric, and intestinal digestion and dialysis (potential uptake); (methanol extract of sprouts). The data presented in this figure consist of average quantities ± *SD* of three independent samples. Different letters in the bars within each sprout variety represent statistically significant differences (*p* < .05)

The TPC in different fractions following in vitro gastrointestinal digestion are shown in Figure 2. The TPC in the methanolic extracts of the sprouts were in the range of 8.34–10.07 mg GAE/g DW, with the RQ sprouts having the highest TPC level while GQ had the lowest level. The behavior of the different quinoa sprout samples differed after the oral phase, with the percentage recovery of polyphenols being within the range of 45.7%–63.5%, relative to the TPC of the methanolic extracts. It can be speculated that only a portion of polyphenols was released during this step. After gastric digestion, the bio‐accessible TPC increased dramatically, recovering 87.6%, 98.0%, 116.7%, 102.5% and 100.3% of the TPC from RQ, WQ, GQ, AQ, and DK, respectively. A similar trend has also been reported from quinoa seeds and grape (Pellegrini et al., 2017; Tagliazucchi, Verzelloni, Bertolini, & Conte, 2010). This may be due to the breaking of bonds between the bioactive compounds and nutrients by the acid medium, which helps to the release polyphenolic compounds from the food matrix (Alminger et al., 2014). The bio‐accessible TPC after intestinal digestion were in the range of 89.6%–124.5%. The TPC bio‐accessibility of the sprouts of the four quinoa genotypes significantly (*p* < .05) increased after the intestinal phase. In contrast, the TPC bio‐accessibility of djulis decreased significantly (*p* < .05). Tagliazucchi et al. (2010) also reported that phenolic compounds continued to be released during intestinal digestion. Polyphenols bound to vegetal matrices, in the form of esters, glycosides, or polymers, cannot be absorbed (da Silva Haas et al., 2019). However, other studies have found that phenolic compounds can be released from these matrices during digestion under the action of pepsin, trypsin and the environmental pH, and are then absorbed in the gut (Manach, Williamson, Morand, Scalbert, & Rémésy, 2005; Parada & Aguilera, 2007), which may explain why the polyphenol recovery index increased during the gastric and intestinal phases.

The recoveries of dialyzable polyphenols from RQ, WQ, GQ, AQ, and DK were 7.4%, 9.4%, 10.9%, 9.9%, and 8.5%, respectively. These results indicated that almost 90% of the polyphenols could not be absorbed by the intestines. Gunathilake et al. (2018) also reported that the dialyzable polyphenols of six edible green leaves had recoveries that varied between 3.06% and 12.30% relative to their fresh leaves. The polyphenols present in the intestinal digesta were not all available for uptake, and the absorption of polyphenols took place via passive diffusion across the epithelial cell barrier of the small intestine. Therefore, the low rates of dialysis might be due to the molecular configuration of the different bioactive molecules and the multiple interactions that occur between bioactive compounds and complex dietary components, all of which determine the absorption of these compounds by the gut (Manach et al., 2005; Mosele, Macià, Romero, & Motilva, 2016).

### 3.2 Effect of in vitro gastrointestinal digestion on the TFC

**FIGURE 3:**
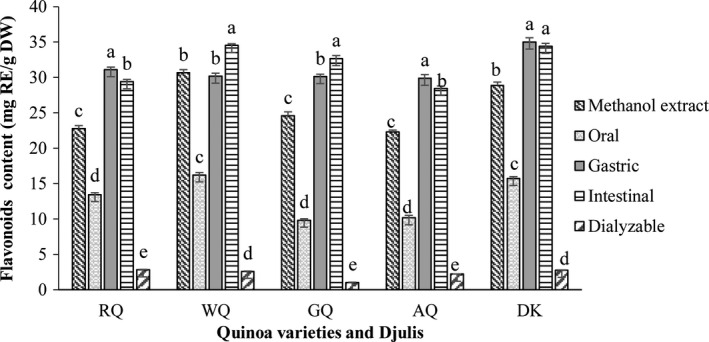
Total flavonoid content of sprouts subjected to simulated in vitro oral, gastric, and intestinal digestion and dialysis (potential uptake); (methanol extract of sprouts). The data presented in this figure consist of average quantities ± *SD* of three independent samples. Different letters in the bars within each sprout represent statistically significant differences (*p* < .05)

The TFC obtained after the oral, gastric, and intestinal phases, and the dialysis and methanol extractions are presented in Figure 3. The TFC in the methanolic extracts of the sprout samples were in the range of 22.29–30.67 mg RE/g DW and the highest TFC was reported in WQ. The pattern of flavonoid release was similar to the polyphenols. Compared to the methanolic extracts, the percentage of flavonoid recovered after the oral phase was in the range of 40.1%–59.1%. The TFC obtained after the gastric and intestinal phases were significantly higher (*p* < .05) than those obtained after the oral phase. The level of bio‐accessible flavonoid detected after the gastric and intestinal phases was higher than those detected by methanol extraction, except WQ had lower values (98.4%) in the gastric phase. There were significant differences among the quinoa sprout samples after the intestinal phase, with WQ and GQ showing a significant increase in the TFC recovery index, while RQ and AQ showed a contrary result. There was no significant difference between the gastric and intestinal phases in the DK sprout. These phenomena could be related to different chemical transformations, under which the flavonoid content would increase following the action of intestinal enzymes on the residual matrix, but the flavonoid may also experience degradation or isomerization in the presence of oxygen and/or transition‐metal ions under near‐neutral conditions (Alminger et al., 2014). The dialyzable flavonoid content of the studied sprout samples was much lower than during the intestinal phase, with the highest dialyzable content obtained from RQ (12.4%), while the lowest content was seen in GQ (4.2%). A similar decreasing trend has also been reported from six types of edible green leaves and broccoli inflorescences (Gunathilake et al., 2018; Vallejo, Gil‐Izquierdo, Pérez‐Vicente, & García‐Viguera, 2004

). This may be due to the low stability of flavonoids and the range of different interactions that they have with other food matrix compounds (Ortega, Macià, Romero, Reguant, & Motilva, 2011).

### 3.3 Effect of in vitro digestion on the individual phenolic compounds

The results obtained from the phenolic profile characterization following methanol extraction are presented in Table 1. Among the methanol extract samples, AQ showed the highest concentration of total phenolic compounds, while the lowest concentration was observed in WQ. In the methanol extraction fractions, the main phenolic acids were p‐coumaric acid, ferulic acid, isoferulic acid, vanillic acid, and caffeic acid, while the contents of syringic acid, protocatechuic acid, and p‐Hydroxybenzoic acid were lower. Gawlik‐Dziki et al. (2013) have previously analyzed the gallic acid, p‐hydroxybenzoic acid, chlorogenic acid, vanillic acid, syringic acid, p‐coumaric acid, ferulic acid, sinapinic acid, benzoic acid, and o‐coumaric acid contents of chemical (ethanolic) quinoa leaf extracts, and the main phenolic acids recorded were ferulic, sinapinic, and gallic acids. In addition, Złotek et al. (2019) also analyzed p‐hydroxybenzoic acid, vanillic acid, p‐coumaric acid, salicylic acid, and ferulic acid contents of methanol extracts from quinoa leaves and sprouts, and the main phenolic component was ferulic acid.

**TABLE 1:** HPLC analysis of the polyphenolic profile of quinoa sprouts following methanol extraction (µg/g DW)

Sample/Stage	RQ	WQ	GQ	AQ	DK
Methanol extraction					
Protocatechuic acid	46.84 ± 0.19c	55.75 ± 0.26b	46.27 ± 0.73c	47.75 ± 1.66c	62.95 ± 1.56a
p‐Hydroxybenzoic acid	47.88 ± 0.86b	32.54 ± 0.91c	16.95 ± 0.62e	28.39 ± 1.34d	73.02 ± 1.13a
Vanillic acid	192.81 ± 3.82b	176.98 ± 2.39c	191.48 ± 1.82b	188.85 ± 2.39b	233.60 ± 7.54a
Caffeic acid	152.38 ± 2.07c	186.44 ± 4.11b	129.38 ± 2.37d	83.58 ± 3.96e	212.80 ± 2.68a
Syringic acid	96.28 ± 1.86b	60.88 ± 3.09d	76.53 ± 2.52c	41.62 ± 1.59e	193.79 ± 4.63a
p‐Coumaric acid	186.54 ± 5.12e	292.68 ± 7.32c	2,648.62 ± 24.20a	1,616.16 ± 21.33	245.90 ± 3.54d
Ferulic acid	120.55 ± 3.15c	142.50 ± 3.15c	125.46 ± 3.91c	2,823.42 ± 29.01a	452.69 ± 20.98b
Isoferulic acid	1,988.87 ± 19.99a	1,649.97 ± 27.75b	1,380.11 ± 14.54c	301.24 ± 7.58e	1,252.03 ± 16.40d
Total	2,832.14 ± 34.62c	2,597.75 ± 12.16e	4,614.80 ± 12.16b	5,131.00 ± 60.40a	2,726.78 ± 24.58d

Note

Data are the means ± *SD* (*n* = 3), different letters within the same column indicate significant differences between different sprout varieties at the 0.05 level.

The results of individual phenolic acid profiles of the oral, gastric, and intestinal samples are presented in Table 2. After digestion, the main phenolic acids were vanillic acid, caffeic acid, and syringic acid. All the studied sprout samples had the lowest amounts of individual phenolic compounds during the intestinal phase. Similarly, Pellegrini et al. (2017) also found that intestinal samples had the lowest concentration of total phenolic compounds. These results confirm that both acid and alkaline hydrolysis during the digestion phases seems to have a strong effect on the stability of phenolic compounds. Polyphenols are more stable at acidic pH and highly sensitive to the mild alkaline conditions in the intestinal phase, and a large proportion of these compounds can be transformed before absorption, thus explaining the lowest bio‐accessibility values during the intestinal phase (Bermúdez‐Soto, Tomás‐Barberán, & García‐Conesa, 2007; Gullon, Pintado, Fernández‐López, Pérez‐Álvarez, & Viuda‐Martos, 2015; Tagliazucchi et al., 2010). The highest total phenolic content of each digestion phase was observed in GQ sprout, whereas the lowest total phenolic content was seen in WQ sprout.

**TABLE 2:** HPLC results obtained from the oral, gastric, and intestinal samples (µg/g DW)

Sample/Stage	RQ	WQ	GQ	AQ	DK
Oral					
Protocatechuic acid	13.81 ± 0.33b	6.54 ± 0.33c	tr	18.05 ± 0.51a	tr
p‐Hydroxybenzoic acid	nd	tr	tr	tr	3.03 ± 0.38
Vanillic acid	810.60 ± 12.97a	56.89 ± 3.82d	806.87 ± 9.48a	479.47 ± 13.08b	435.03 ± 10.02c
Caffeic acid	108.43 ± 4.37b	117.96 ± 2.32a	109.78 ± 4.93b	83.74 ± 1.04c	110.77 ± 3.30b
Syringic acid	1,052.50 ± 11.42c	760.63 ± 2.62d	1,677.94 ± 14.54a	1,299.37 ± 4.32b	581.68 ± 10.30e
p‐Coumaric acid	nd	tr	nd	nd	tr
Ferulic acid	141.26 ± 3.53b	107.80 ± 2.03c	112.98 ± 2.85c	nd	244.54 ± 5.04a
Isoferulic acid	nd	nd	nd	nd	nd
Total	2,126.59 ± 32.40b	1,049.83 ± 4.59e	2,707.57 ± 26.91a	1,880.63 ± 17.91c	1,375.05 ± 1.68d
Gastric					
Protocatechuic acid	8.02 ± 0.60c	23.86 ± 0.36a	tr	15.60 ± 0.57b	tr
p‐Hydroxybenzoic acid	nd	tr	tr	tr	1.92 ± 0.31
Vanillic acid	704.91 ± 22.58a	52.77 ± 1.47d	708.27 ± 17.34a	516.09 ± 6.28c	655.94 ± 15.46b
Caffeic acid	136.01 ± 2.85b	87.90 ± 1.33d	100.27 ± 1.95c	85.33 ± 1.46d	251.93 ± 2.41a
Syringic acid	846.53 ± 10.34c	783.42 ± 14.36d	1,650.29 ± 16.90a	1,586.87 ± 27.79b	638.04 ± 9.50e
p‐Coumaric acid	nd	tr	nd	nd	nd
Ferulic acid	206.45 ± 3.26b	109.84 ± 2.83d	131.36 ± 6.21c	nd	341.93 ± 4.43a
Isoferulic acid	nd	nd	nd	nd	nd
Total	1,901.92 ± 17.69c	1,057.79 ± 14.57d	2,613.56 ± 8.75a	2,203.89 ± 32.23b	1,911.33 ± 28.30c
Intestinal					
Protocatechuic acid	tr	53.09 ± 0.78	tr	5.95 ± 0.39	tr
p‐Hydroxybenzoic acid	tr	tr	tr	nd	4.50 ± 0.28
Vanillic acid	314.13 ± 7.01c	36 ± 0.74e	561.56 ± 10.38a	293.09 ± 3.53d	398.75 ± 12.05b
Caffeic acid	88.51 ± 0.88c	133.53 ± 1.62b	157.44 ± 1.61a	61.45 ± 2.90d	132.93 ± 4.16b
Syringic acid	852.25 ± 5.61c	506.49 ± 9.08d	1,609.73 ± 16.11a	1,157.38 ± 11.42b	465.57 ± 7.41e
p‐Coumaric acid	nd	5.01 ± 0.81	nd	nd	nd
Ferulic acid	134.24 ± 3.37b	190.23 ± 1.91a	186.22 ± 8.47a	nd	181.81 ± 5.41a
Isoferulic acid	nd	nd	nd	nd	nd
Total	1,389.32 ± 3.87c	924.35 ± 8.38e	2,514.95 ± 19.08a	1,517.88 ± 18.24b	1,183.55 ± 29.23d

Note

Data are the means ± *SD* (*n* = 3), different letters within the same column indicate significant difference between different sprout varieties at the 0.05 level; Total, sum of detected compounds.

Abbreviations: nd, not detected; tr, trace.

The potential to take up phenolic acid after the simulated in vitro digestion phase was observed to have decreased significantly (Table 3). Among the dialysis samples, GQ showed the highest concentrations of total phenolic acids, while WQ had the lowest concentrations of total phenolic acid. Notably, p‐hydroxybenzoic acid, p‐coumaric acid, and isoferulic acid were not detected in dialysis samples. Therefore, we hypothesized that some individual phenolic compounds may degrade or transform during digestion.

**TABLE 3:** HPLC results obtained from the dialysis samples (µg/g DW)

Sample/Stage	RQ	WQ	GQ	AQ	DK
Dialysis					
Protocatechuic acid	tr	3.99 ± 0.09	tr	1.64 ± 0.06	tr
p‐Hydroxybenzoic acid	nd	nd	nd	nd	tr
Vanillic acid	29.41 ± 0.98b	2.33 ± 0.06d	35.23 ± 0.54a	27.33 ± 0.45c	28.02 ± 0.40b
Caffeic acid	8.85 ± 0.30b	9.15 ± 0.30b	11.26 ± 0.59a	6.96 ± 0.02c	6.72 ± 0.13c
Syringic acid	83.36 ± 0.45c	40.81 ± 0.17e	113.31 ± 0.85a	108.61 ± 3.17b	46.18 ± 0.75d
p‐Coumaric acid	nd	tr	nd	nd	nd
Ferulic acid	nd	nd	12.91 ± 0.30	nd	14.77 ± 0.34
Isoferulic acid	nd	nd	nd	nd	nd
Total	121.61 ± 0.22c	55.48 ± 0.48e	172.72 ± 1.10a	144.21 ± 2.79b	95.68 ± 1.41d

Note

Data are the means ± *SD* (*n* = 3), different letters within the same column indicate significant difference between different sprout varieties at the 0.05 level; Total, sum of detected compounds.

Abbreviations: nd, not detected; tr, trace.

### 3.4 Antioxidant capacity

The antioxidant activity of different fractions from the in vitro gastrointestinal digestion procedure is shown in Table 4. The antioxidant capacity measured by DPPH and ABTS significantly increased (*p* < .05) during the gastric stage relative to the oral stage, which may indicate the release of bioactive compounds after digestion of the sprouts. This result paralleled previous studies demonstrating that antiradical power was significantly increased after simulated gastrointestinal digestion (Gawlik‐Dziki, 2012).

**TABLE 4:** Antioxidant activity results obtained from ABTS and DPPH assays

Samples	Methanolic extract	Oral	Gastric	Intestinal	Dialysis
ABTS radical scavenging ability µmol TE/g (DW basis)					
RQ	70.41 ± 0.95a	52.11 ± 0.53d	65.52 ± 0.34d	95.64 ± 0.34a	9.66 ± 0.03a
WQ	61.10 ± 0.55d	47.64 ± 0.69e	61.43 ± 0.25e	93.60 ± 0.17c	9.35 ± 0.03d
GQ	73.66 ± 0.93b	63.31 ± 0.17b	79.86 ± 0.33b	95.08 ± 0.17b	9.41 ± 0.02c
AQ	70.68 ± 0.95c	60.72 ± 0.25c	71.86 ± 0.53c	95.42 ± 0.17ab	9.48 ± 0.02b
DK	84.91 ± 0.70a	65.90 ± 0.25a	88.58 ± 0.24a	95.80 ± 0.19a	9.64 ± 0.02a
DPPH radical scavenging ability µmol TE/g (DW basis)					
RQ	30.25 ± 0.08a	12.97 ± 0.10a	15.95 ± 0.06d	18.88 ± 0.07a	1.14 ± 0.02b
WQ	16.78 ± 0.18e	6.95 ± 0.11d	13.05 ± 0.17e	13.66 ± 0.08e	0.58 ± 0.02d
GQ	28.34 ± 0.08d	9.00 ± 0.34c	17.88 ± 0.03b	18.19 ± 0.06b	1.21 ± 0.01a
AQ	28.61 ± 0.10c	13.10 ± 0.11a	16.50 ± 0.19c	16.69 ± 0.13d	1.11 ± 0.00b
DK	29.75 ± 0.16b	11.49 ± 0.11b	18.28 ± 0.06a	17.73 ± 0.10c	1.03 ± 0.04c

Note

Data are the means ± *SD* (*n* = 3), different letters within the same column indicate significant differences between different sprout varieties at the 0.05 level.

In the present work, the ABTS antioxidant activity of the dialysis samples was in the range of 9.35–9.66 µmol TE/g DW, which was only one‐tenth of the intestinal digested samples (93.60–95.80 µmol TE/g DW). Similarly, the DPPH antioxidant activity of the dialysis samples was significantly lower than that of the intestinal digested samples as well as methanolic extract samples (*p* < .05). The results are consistent with previous studies that reported the antioxidant activity of polyphenols extracts from coffee beans obtained after simulated absorption was significantly lower than that of simulated digestion samples (Cheng et al., 2019; Dziki et al., 2015).

**FIGURE 4:**
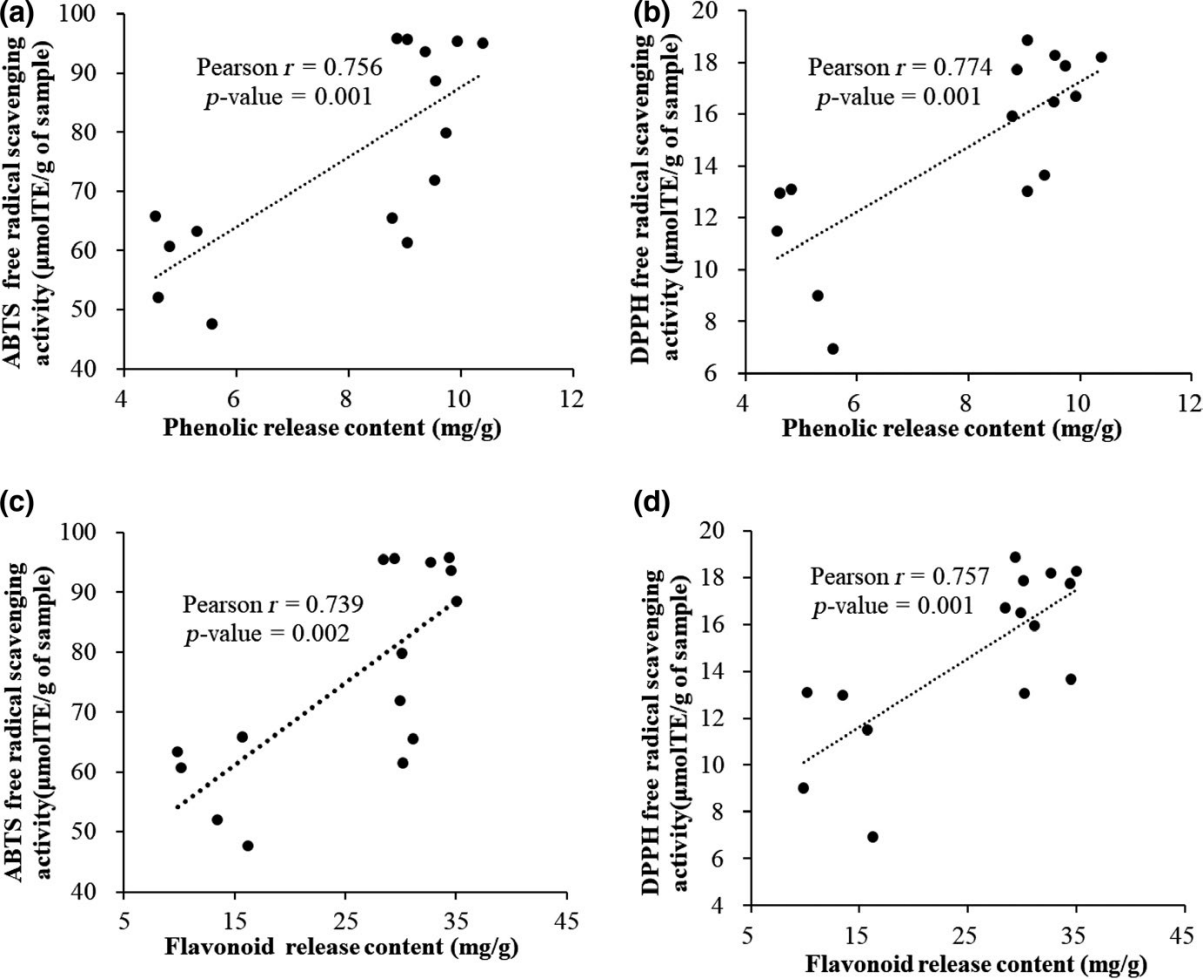
(a) correlation between the phenolic release content and ABTS free radical scavenging activity; (b) correlation between the phenolic release content and DPPH free radical scavenging activity; (c) correlation between the flavonoid release content and ABTS free radical scavenging activity; (d) correlation between the flavonoid release content and DPPH free radical scavenging activity

There was a positive correlation between free radical scavenging activity and TPC released from the different digestion steps (ABTS: *r* = .756, *p* = .001; DPPH: *r* = .774, *p* = .001), as well as between free radical scavenging activity and TFC released from the different digestion steps (ABTS: *r* = .739, *p* = .002; DPPH: *r* = .757, *p* = .001) (Figure 4). Similarly, previous study found that there was a positive correlation between total polyphenols of plant material and free radical scavenging activity (Gunathilake & Ranaweera, 2016). Based on the literature, the antioxidant activity of a polyphenol is related to its chemical structure. For example, polyphenol aglycones display a higher antioxidant activity than their glycosides (Kamiloglu, Pasli, Ozcelik, & Capanoglu, 2014). The type and quantity of polyphenolic compounds are related to the scavenging of free radicals (Cao, Sofic, & Prior, 1997). Further, the interaction of polyphenols with other dietary molecules released from food matrices during the digestion process is known to affect polyphenol solubility and availability, and thus affect the antioxidant potential (Bouayed, Hoffmann, & Bohn, 2011).

## 4 CONCLUSIONS

The present study suggests that polyphenols and flavonoids of quinoa and djulis sprouts are progressively released and made available for absorption in an in vitro model of gastrointestinal digestion. Compared to the oral phase, the recovery index of polyphenols and flavonoid significantly increased (*p* < .05) in the gastric and intestinal phase. However, the final potential uptake of polyphenols and flavonoids after simulated in vitro digestion was significantly lower (*p* < .05) than the amount originally available in the leaves. Some individual phenolic compounds might degrade or transform during digestion. There was a positive correlation between the antioxidant activity and TPC or TFC released from different digestion steps. Further studies should be undertaken to support the findings of this study and to better understand the factors that affect the bio‐accessibility of bioactive compounds in quinoa and djulis sprouts.

## CONFLICT OF INTEREST

The authors declare no conflict of interest.

## ETHICAL APPROVAL

Neither animal nor human testing was involved in this study.
